# Thermal changes in guidewires used during endoscopic retrograde cholangiopancreatography during electrical conductance: Ex-vivo safety experiment

**DOI:** 10.1055/a-2536-7997

**Published:** 2025-03-14

**Authors:** Rahul Karna, Nicholas M McDonald, Jacob Weiner, Théodon I Netoff, Stuart K Amateau

**Affiliations:** 1 14400 Division of Gastroenterology, Hepatology & Nutrition, University of Minnesota Medical Center, Minneapolis, United States; 2 6559 Department of Medicine, Division of Gastroenterology, Hepatology & Nutrition, Thomas Jefferson University, Philadelphia, United States; 3 1259 Department of Biomedical Engineering, University of Minnesota, Minneapolis, United States

**Keywords:** Pancreatobiliary (ERCP/PTCD), ERC topics, Quality and logistical aspects, Performance and complications

## Abstract

Risks of pancreaticobiliary tissue damage secondary to electrical conduction along cannulation wires in the vicinity of electrocautery have rarely been independently studied and remain mostly a theoretical issue. We aimed to evaluate heat generated by commercially available guidewires in the setting of currents used for sphincterotomy to assess their safety during endoscopic procedures. We tested heat generation from 11 common guidewires used in endoscopic retrograde cholangiopancreatography using an electrosurgical generator in combination with a thermocouple temperature probe in an ex vivo model. Thermal changes during electrical conductance were nominal except for minimally wrapped 0.018” wires where the maximum changes in temperature were 4.9°C with ENDO CUT Q settings and 6.8°C with ENDO CUT I settings. Also, the Glidewire 0.018” and the Visiglide 0.025” produced visible sparks along their distal ends with defects in insulation found later under microscopic evaluation. In our ex-vivo study, minimal heat was generated via electrical conductivity despite direct current, suggesting negligible risk of thermal injury during sphincterotomy.

## Introduction

**Fig. 1:**
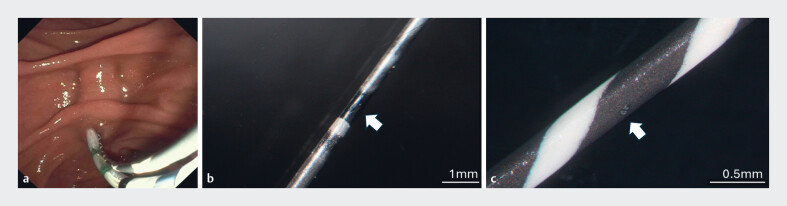
**a** Dual wire cannulation demonstrating the possibility of the cutting wire contacting the alternate guidewire. **b** Magnified view (13.6X) of Glidewire 0.018” shows damage in the form of discoloration noted ~6.5 inches from the tip of the guidewire. **c** Magnified (40X) examination of Visiglide 0.025” shows microlesions observed approximately 3.5 inches from tip of the wire and approximately 5.08 mm into the hydrophilic coating.

Endoscopic retrograde cholangiopancreatography (ERCP) is one of the most widely performed endoscopy procedures across the world 1 . Despite technological advances, improved operator expertise and recognition of high-risk patients, ERCP is associated with a variety of short- or long-term complications 2 . Guidewires are universally used during ERCP to direct deep pancreaticobiliary cannulation. Frequently, dual wire cannulation technique is required, that being use of a second wire to cannulate the desired duct following initial cannulation of the other. Endoscopists then often leave both guidewires in place during sphincterotomy. In this scenario, the wire maintaining access to the alternative duct may come into contact with the cutting wire of the sphincterotome, in theory allowing electrical conduction and thermal injury along the length of the contacted wire ( Fig. 1 **a** ).

Similarly, during endoscopic ampullectomy for noninvasive ampullary adenoma, a guidewire can be utilized to maintain pancreatic duct access and to allow for pancreatic duct stenting after ampullectomy 3 4 5 . With a wire-guided approach during ampullectomy, the snare often contacts the guidewire, allowing for the possibility of electrical conduction along the length of the wire within the pancreatic duct. With the increase in demand and popularity of therapeutic endoscopic procedures involving wire-guided approaches, we sought to evaluate the electrical conductivity of a number of commonly used guidewires, through an ex-vivo experimental design.

## Materials and methods

We tested heat generation of 11 wires commonly used in ERCP: Glidewire 0.018” (Terumo Interventional Systems, Somerset, New Jersey, United States), Novagold 0.018” (Boston Scientific, Marlborough, Massachusetts, United States), Road Runner 0.018” (Cook Medical, Bloomington, Indiana, United States), Tracer Metro 0.021” (Cook Medical, Bloomington, Indiana, United States), Glidewire 0.025” (Terumo Interventional Systems, Somerset, New Jersey, United States), Visiglide 0.025” (Olympus, Center Valley, Pennsylvania, United States), Revowave 0.025” (Olympus, Center Valley, Pennsylvania, United States), Jagwire Revolution 0.025” (Boston Scientific, Marlborough, Massachusetts, United States), Glidewire 0.035” (Terumo Interventional Systems, Somerset, New Jersey, United States), Jagwire 0.035” (Boston Scientific, Marlborough, Massachusetts, United States), and Hydra Jagwire 0.035” (Boston Scientific, Marlborough, Massachusetts, United States) ( Table 1 ). A polypectomy snare was connected to the wire. An electrosurgical generator (ERBE, Marietta, Georgia, United States) with settings ENDO CUT Q (effect 2, cut duration 2, cut interval 3) and ENDO CUT I (effect 2, cut duration 2, cut interval 3) was utilized as the energy source.

**Table:** **Table 1** Thermal changes produced by commercially available guidewires during electrical conductance via surgical generator in our experiment.

Wire	Diameter (inches)	Starting temp ENDO CUT Q	Final temp ENDO CUT Q	Delta temp ENDO CUT Q	Presence of spark	Starting temp ENDO CUT I	Final temp ENDO CUT I	Delta temp ENDO CUT I	Presence of spark
Glidewire (Terumo Interventional Systems, Somerset, New Jersey, United States)	0.018	18.0 °C	18.0°C	0.0°C	Yes	17.5°C	17.4°C	-0.1°C	Yes
Novagold (Boston Scientific, Marlborough, Massachusetts, United States)	0.018	18.2°C	21.5°C	3.3°C	No	18.5°C	23.4°C	4.9°C	No
Road Runner (Cook Medical, Bloomington, Indiana, United States)	0.018	17.7°C	22.0°C	4.3°C	No	18.0°C	24.8°C	6.8°C	No
Tracer Metro (Cook Medical, Bloomington, Indiana, United States)	0.021	17.9°C	17.9°C	0.0°C	No	17.8°C	17.8°C	0.0°C	No
Glidewire (Terumo Interventional Systems, Somerset, New Jersey, United States)	0.025	17.3°C	17.3°C	0.0°C	No	17.2°C	17.2°C	0.0°C	No
Visiglide (Olympus, Center Valley, Pennsylvania, United States)	0.025	17.4°C	17.3°C	-0.1°C	Yes	17.8°C	17.7°C	-0.1°C	Yes
RevoWave (Olympus, Center Valley, Pennsylvania, United States)	0.025	18.2°C	18.2°C	0.0°C	No	18.0°C	18.0°C	0.0°C	No
Jagwire Revolution (Boston Scientific, Marlborough, Massachusetts, United States)	0.025	17.9°C	17.9°C	0.0°C	No	17.7°C	17.5°C	-0.2°C	No
Glidewire (Terumo Interventional Systems, Somerset, New Jersey, United States)	0.035	18.1°C	18.2°C	0.1°C	No	17.6°C	17.7°C	0.1°C	No
Jagwire (Boston Scientific, Marlborough, Massachusetts, United States)	0.035	18.1°C	18.1°C	0.0°C	No	18.1°C	18.0°C	-0.1°C	No
Hydra Jagwire (Boston Scientific, Marlborough, Massachusetts, United States)	0.035	17.8°C	17.8°C	0.0°C	No	17.8°C	17.8°C	0.0°C	No

**Fig. 2:**
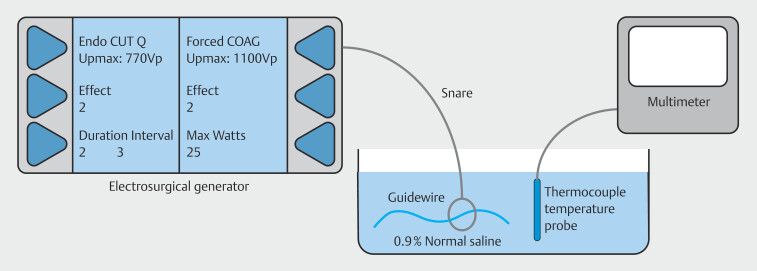
An illustration showing the benchtop model used in the study.

A thermocouple temperature probe (Grainger, Lake Forest, Illinois, United States) was submerged in a basin containing a 0.9% saline solution (initial temperature: 17.3°C). The probe was connected to a Fluke 15B+ digital multimeter (Fluke Corporation, Everett, Washington, United States) and the distal ends of each guidewire were submerged in the basin immediately adjacent to the probe tip. Following guidewire placement, an electrical current was applied for 10 seconds to the wire, and the resulting temperature change proximal to the guidewire tip was recorded. Fig. 2 illustrates the experimental setup of our study.

During our experiment, we noted that two of the wires generated a visible spark along the distal ends of the wire when initial current was applied. To assess this further, we also applied current to all of the wires outside of the basin of saline to evaluate for a visible spark. We then evaluated the wires visually under a microscope (Microscope: Zeiss Discovery Lumar V12 Fluorescence SteREO Microscope with two KL 2500 LCDs) to assess for visible defects.

## Results

### Thermal effects produced during the experiment

Eleven wires were tested for heat generated and for presence of a spark from electrical conductance via an electrical surgical generator. Only the two uninsulated 0.018” wires demonstrated any significant temperature change; 4.3°C (7.9°F) using ENDO CUT Q and 6.8°C (12.2°F) using ENDO CUT I with the Road Runner wire and 3.3°C (5.9°F) using ENDO CUT Q and 4.9°C (8.8°F) using ENDO CUT I with the NovaGold wire. No changes in temperature were recorded regardless of the cut settings with any of the other nine wires, including an uninsulated 0.018 Glidewire. During the experiment, two wires, the 0.018” Glidewire and the 0.025” Visiglide, produced visible spark along their distal ends, whereas the others were without any visible findings.

### Microscopic examination of wire

Wires were assessed macroscopically and microscopically to assess for defects after the experiment. There was one circular defect macroscopically visible on Glidewire 0.018” with an area of missing coating located approximately 1.70” from the tip of the wire. Upon magnified view (13.6X), discoloration was also noted approximately 6.5” from the tip of the guidewire ( Fig. 1 **b** ). Multiple small relatively deep defects were macroscopically visible on Visiglide 0.025”. Upon magnified (40X) examination, these microlesions were observed approximately 3.5” from tip of the wire and approximately 5.08 mm into the hydrophilic insulated coating ( Fig. 1 **c** ).

## Discussion

The safety of guidewires used in advanced endoscopy has rarely been studied. A similar study from Young and Darwin showed that with four wires (Jagwire 0.025”, Jagwire 0.035”, Hydra Jagwire 0.035” [at that time Microvasive, Natick, Massachusetts, United States], and a defunct Zebra 0.035” (Melville, New York, United States), electrical current was not appreciably conducted to cause local tissue injury 1 . However, the potential for many of the current commercially available wires used in ERCP had not been reported previously.

Numerous variables such as inch diameter, length, and tip angle are frequently taken into consideration when choosing a wire for selective duct cannulation. This study reveals that insulation should also be considered because two uninsulated wires allowed for temperature change. This temperature change reflects conduction of current and these changes were relatively minimal. It should be noted that wire coating is not equivalent to wire insulation. Both the 0.018” Glidewire and the 0.018” Novagold have spray hydrophilic coatings without insulation and the Road Runner has neither. The prescriptive documentation associated with these wires clearly states that their use should be limited to selective cannulation. However, this ex vivo demonstration reveals that even when the current is applied directly to the wire through use of a snare, temperature change was at most minimal, and possibly negligible if current from a sphincterotome cutting wire was not in direct contact with the guidewire.

To our surprise, we noted two wires (Glidewire 0.018” and Visiglide 0.025”) generated electrical sparks down their distal ends. Macroscopic and microscopic inspection of these wires revealed defects in their coatings that allow these abrupt electrical discharges. This was repeated and replicated with similar wires. It is appreciated, however, that this may not reflect a gross design flaw with these particular wires, but rather, a defect unique to the particular batches studied.

Given these results, overall risk of thermal injury from electrical conductance appears minimal during standard endoscopic cannulation and sphincterotomy, even when using uninsulated wires. However, in situations where the cutting wire of the sphincterotome may come into direct contact with an uninsulated wire, such as with over-the-wire ampullectomy or dual wire cannulation, the physicians should recognize that electrical conduction will result in temperature changes within the duct, albeit minimal. One important caveat is the potential for tissue damage due to electrical sparks secondary to microscopic defects in various insulated coatings during direct application of electrocautery to the wire. Limitations of our study included the assumption of an undamaged wire and the ex-vivo nature of the study, and thus, we could not assess the impact of temperature change on the mucosal surface.
